# A miniaturized iodine value assay for quantifying the unsaturated fatty acid content of lipids, lipid mixtures, and biological membranes

**DOI:** 10.1002/lipd.12438

**Published:** 2025-02-23

**Authors:** Maria Monserrat Roman‐Lara, Katie J. Chong, Roslyn M. Bill, Alan D. Goddard

**Affiliations:** ^1^ Aston Institute for Membrane Excellence and School of Biosciences, College of Health and Life Sciences Aston University Birmingham UK; ^2^ Energy and Bioproducts Research Institute, College of Engineering and Physical Sciences Birmingham UK

**Keywords:** cell membrane, colorimetry, Iodine Value, lipid unsaturation, Wijs reagent

## Abstract

Various methods exist for identifying and quantifying lipid unsaturation, including mass spectrometry and Raman spectroscopy. A disadvantage of these existing approaches is the need for sophisticated equipment and software, placing them beyond the means of many laboratories. The iodine value (IV) is a colorimetric unsaturation index; however, it uses iodine monochloride, a hazardous chemical, and considerable amounts of sample. Here, we demonstrate the first use of a miniaturized IV method that requires only milliliter quantities of hazardous chemicals and sample sizes such that it is feasible to assay biological membranes. Briefly, lipids are exposed to iodine monochloride, resulting in the replacement of unsaturated bonds with di‐halogenated single bonds. Potassium iodide then reacts with unreacted iodine monochloride forming I_2_, which is quantified through titration with sodium thiosulfate. To demonstrate the biological relevance of our assay, membrane lipids of *Escherichia coli* grown at 30, 37, and 42°C were analyzed, with IV increasing as temperature decreased, as would be expected. Importantly, multiple samples could be rapidly and simultaneously analyzed in a reproducible assay that did not require sophisticated equipment or data analysis methods. Our miniaturized IV assay will benefit laboratories with limited access to sophisticated equipment and enable the rapid determination of lipid unsaturation in milligram‐scale samples.

## INTRODUCTION

The unsaturation of lipids is fundamental in determining their biological activity, and it is often desirable to determine the degree of unsaturation of biological samples from a variety of diverse sources. Numerous techniques are available to detect and measure lipid unsaturation. Thin‐layer chromatography (TLC) is a standard method (Singh et al., 2018); however, it cannot give exact quantification, and if the TLC plate is exposed to atmospheric oxygen, the lipids can oxidize (Shanta & Napolitano, 1998). Other chromatographic methods include gas (GC) and liquid (LC) chromatography, which, when coupled to mass spectrometry (MS) (Wolrab et al., 2022), can offer high precision, molecular specificity, and sensitivity. However, as many lipids have similar masses, it can be challenging to determine the nature of each molecular species precisely. These methods also involve several time‐consuming steps and may not be suitable for high‐throughput analyses (Forfang, 2017). Dielectric spectrometry (Patel et al., 2019), nuclear magnetic resonance spectroscopy (NMR) (Hernandez Cravero et al., 2024), fluorescence, and Raman spectroscopy (Patel et al., 2019) can estimate lipid unsaturation and quantify saturated and unsaturated fatty acids in high‐throughput formats. However, these approaches (as well as mass spectrometry) require advanced equipment, specialized software, and trained personnel. In addition, the large, complex datasets generated can make data analysis challenging. In the spirit of making science accessible and ensuring researchers can determine the unsaturation status of samples with minimal specialist equipment, we revisit the classic iodine value (IV) assay with the aspiration of miniaturizing it to allow application to biological samples that can be limited in mass.

IV is defined as the number of centigrams of iodine absorbed per gram of sample. Iodine (or other halogens) binds to carbon‐carbon double bonds, so it can be used to quantify the number of unsaturated bonds in fats or oils. IV determination involves the reaction of a halogenating reagent (Hanus or Wijs solution) with the unsaturated double bonds over 30 min. The Wijs method is more common (Pardeshi, 2020). In this process, di‐halogenated single bonds are produced by the iodine monochloride (ICl) reaction with unsaturated bonds. Potassium iodide solution (KI) is then added, causing any unreacted ICl to form molecular iodine (I_2_), leading to the solution becoming dark brown. I_2_ is quantified through titration with sodium thiosulfate (Na_2_S_2_O_3_); sodium iodide (NaI) is generated, causing the solution to become colorless. The quantity of sodium thiosulfate required to convert the dark solution to a colorless one is employed to calculate the IV of the sample (Equation 1). A negative control (blank), which includes all the reagents used except for the oil or fat, is included. In this quantitative assay, a higher IV indicates a bigger level of unsaturation.

IV formula:

$$\mathrm{IV}=\frac{\left(B-S\;\right)\times N\times 126.9\times 100\times 10^{-3}}{W}$$

(1) $$\mathrm{IV}=\frac{\left(B-S\;\right)\times N\times 12.69}{W}$$

where *B* = milliliters of sodium thiosulfate (Na_2_S_s_O_3_) used for the negative control (blank); *S* = milliliters of Na_2_S_s_O_3_ used to assay the sample; *N* = 0.1 = sodium thiosulfate normality; 126.9 = molecular mass of iodine; *W* = weight of the sample used in grams.

The IV is an established analytical method for measuring the total unsaturation of fats and oils. IV has found extensive utility in quality control processes, particularly in the hydrogenation of industrial products such as biodiesel and plant oils (Knothe, 2002). It is recognized by the American Oil Chemists' Society (AOCS) (Kyriakidis & Katsiloulis, 2000) and, as such, is a standard approach in these fields, but has limited applicability in wider bioscience due to the requirement for relatively large sample masses and a low throughput approach. Despite the well‐established IV assay, several researchers have made improvements, so it is faster (Pardeshi, 2020) or can be deployed as a smartphone camera‐based colorimetric method (Peamaroon et al., 2021). Notably, ICl and chloroform (CH_3_Cl is used to solubilize the test sample) are hazardous chemicals requiring safe disposal. It is, therefore, essential to minimize their use for economic, environmental, and health reasons (Aboagye et al., 2021). Initially designed for large‐scale fats and oils in the food and petrochemical industries on a gram‐scale, the IV's simplicity, simple nature, lack of dependence on complex instrumentation, and compatibility with biological samples' linear make it an attractive option for their analysis if the sample size could be reduced.

To our knowledge, we demonstrate the first use of a miniaturized IV method to calculate unsaturation in cell membrane samples (one of the most limited biological samples). Our method features improved simplicity, allows multiple samples to be investigated at once, requires only a small amount of sample and reagents, and can be semi‐automated using a plate reader. To demonstrate the applicability of this approach, we quantified unsaturation in different lipids and *Escherichia coli* lipid membranes grown at different temperatures. This methodology should be of interest to researchers working on cell membranes in both academia and industry.

## MATERIALS AND METHODS

### Reagents

0.1 M ICl solution (Acros Organics, UK) diluted in acetic acid; 15% KI (Acros Organics, UK); 0.1 N Na_2_S_2_O_3_ (Alfa Aesar, USA); and 2% starch solution (Acros Organics, UK) diluted in water.

### Lipid preparation

14:0 DMPC (1,2‐dimyristoyl‐sn‐glycero‐3‐phosphocholine), 18:1 DOPC (1,2‐dioleoyl‐sn‐glycero‐3‐phosphocholine), 22:0 PC (1,2‐dibehenoyl‐sn‐glycero‐3‐phosphocholine), 22:6 (*cis*) PC (1,2‐didocosahexaenoyl‐sn‐glycero‐3‐phosphocholine), and *E. coli* total lipid extract profile were purchased from Avanti Polar Lipids (USA). Lipids were resuspended in chloroform.

### *E. coli* membranes

*E. coli* (DH5α) from glycerol stocks was used to inoculate 60 mL of LB and cultured at 37°C overnight. Each culture was diluted into 300 mL LB medium and incubated at one of three different temperatures (30, 37, or 42°C), shaking at 180 rpm for 48 h. After incubation, cell pellets were collected by centrifugation (4000 x rpm, 15 min). Using a modified Bligh and Dyer method (Bligh & Dyer, 1959) and a Methyl‐ter‐Butyl Ether (MTBE) extraction method (Matyash et al., 2008), the total bacterial lipids were extracted. The total lipids present in the organic phase were then quantified using the Stewart Assay (Stewart, 1980) and a standard curve from 0 to 50 μg of 1 POPC:1 POPE:1 POPG or 1 POPE:1 POPG:1 Cardiolipin.

### Iodine value assay

**FIGURE 1:**
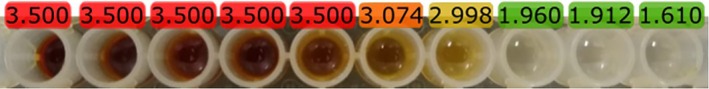
Detection of the loss of color in the miniaturized iodine value assay. The absorbance of wells containing 0.1 M iodine monochloride mixed with 2% starch and 15% KI was measured. Starting from the left, the first well contained no Na_2_S_2_O_3_; the subsequent well to the right was supplemented with 15 μL of 0.1 N Na_2_S_2_O_3_. This process continued, with each successive well receiving an additional 15 μL of 0.1 N Na_2_S_2_O_3_ until the final well had a total of 135 μL of 0.1 N Na_2_S_2_O_3_. The absorbance was quantified at 450 nm, as indicated at the top of each well.

Approximately 1 mg lipid diluted in chloroform was placed in a glass‐coated 96‐well plate. For lipid mixtures, 10 mg of the mixture was diluted in 1 mL chloroform, and 100 μL added per well of a glass‐coated 96‐well plate (Thermo Fischer, USA) so that each well contained 1 mg total lipid. Approximately 40 μL 0.1 M ICl was added to each, followed by a dark, room temperature incubation for 30 min, then 2 μL 2% starch and 20 μL 15% KI were added. A multiwell plate reader (BMG Labtech, Germany) was used to measure the absorbance at 450 nm and dispense sequential 5 μL aliquots of 0.1 N Na_2_S_2_O_3_. Between each aliquot, the plate was shaken for 3 s, and the absorbance was read until the samples were colorless (OD_450_ ≤ 1.99) (Figure 1). Saturated DMPC (Avanti Polar Lipids, USA) was used as a blank. The volume, in microliters, of Na_2_S_2_O_3_ was used to turn the sample solutions and the blank colorless was then applied in the IV formula (Equation 1). All assays were performed in triplicate.

## RESULTS AND DISCUSSION

### Scale‐down of the IV assay

The IV assay involves a titration that transforms a dark solution into a colorless one. The method approved by the AOCS necessitates sufficient sample and solution to ensure that the color change is perceptible to the naked eye. To allow standardization and automation, we tested whether the loss of color could be detected using a spectrophotometer. Initially, a wavelength scan (350–650 nm) of a solution of 0.1 M ICl with 2% starch and 15% KI was titrated with different amounts of 0.1 N Na_2_S_2_O_3_ to determine the optimal wavelength and the optical density (OD) cutoff for a colorless endpoint. An absorbance of <1.99 at 450 nm was chosen as the most sensitive indicator of the solution's colorlessness (Figure 1). The scattering of starch or adding an excess of Na_2_S_2_O_3_ can prevent a lower absorbance from being achieved.

**FIGURE 2:**
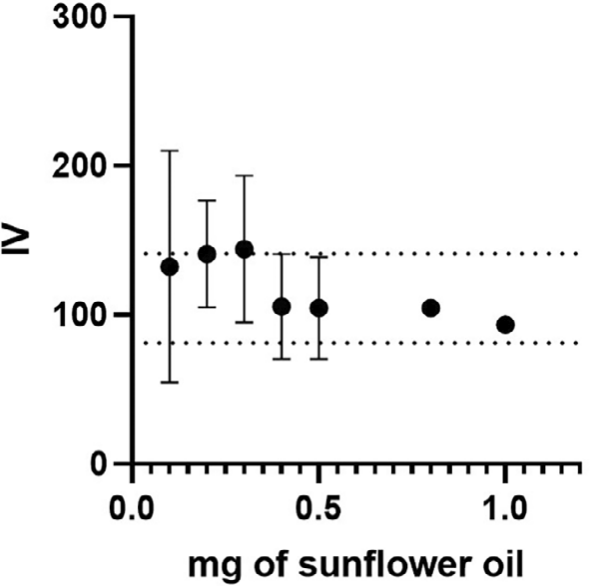
Determination of the iodine value (IV) assay limit of detection. The miniaturized IV assay analyzed different masses of sunflower oil. The reported minimum and maximum IV for sunflower oil are denoted by the dashed lines (*n* = 3, error bars correspond to the SEM; for 0.8 and 1 mg, the SEM are too small to be seen; these are ±4.8 and 3.0, respectively).

After determining the optimal wavelength and optical density cutoff, sunflower oil was used as a known substrate to scale‐down the IV assay and confirm literature values. The miniaturized assay was performed on quantities ranging from 1 to 0.1 mg (Figure 2) to determine the minimum mass of lipid detectable in the assay. Published IV values for sunflower oil are between 81 and 141 (Tiefenbacher, 2017).

**FIGURE 3:**
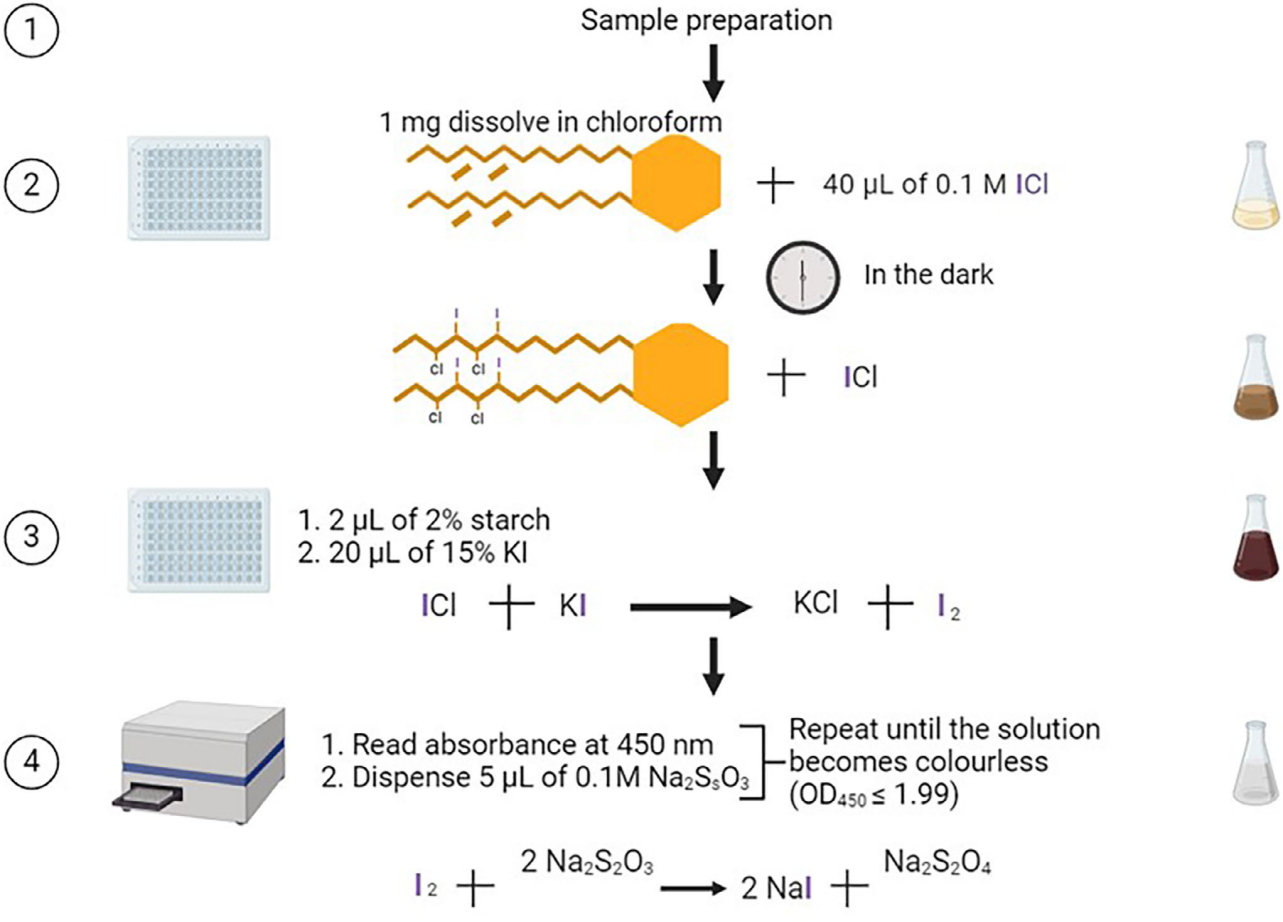
Flowchart of the miniaturized iodine value assay and its reactions. (1) Preparation of the sample. (2) Dissolution of 1 mg sample in chloroform followed by the addition of 40 μL 0.1 M iodine monochloride (ICl) and a 30 min incubation in darkness to facilitate the formation of dihalogenated single bonds. (3) Addition of 2 μL 2% starch plus 20 μL 15% KI to promote the conversion of unreacted ICl to I_2_. (4) Reading of the plate at 450 nm, the addition of 5 μL of 0.1 M Na_2_S_2_O_3_, and shaking. The procedure is repeated until OD reaches ≤1.99, indicating the formation of NaI. The color of the solution at each step is illustrated in the flasks on the right side of the flowchart. Created with Biorender.com.

Although the average IV for all lipid masses tested was within the range of the reported values, the assay's variability increased substantially at lower masses. This demonstrates that the lowest mass of lipid that can be detected reproducibly is 0.5 mg for sunflower oil, but this is likely affected by the degree of unsaturation. The protocol of the miniaturized IV assay is represented schematically in Figure 3.

**FIGURE 4:**
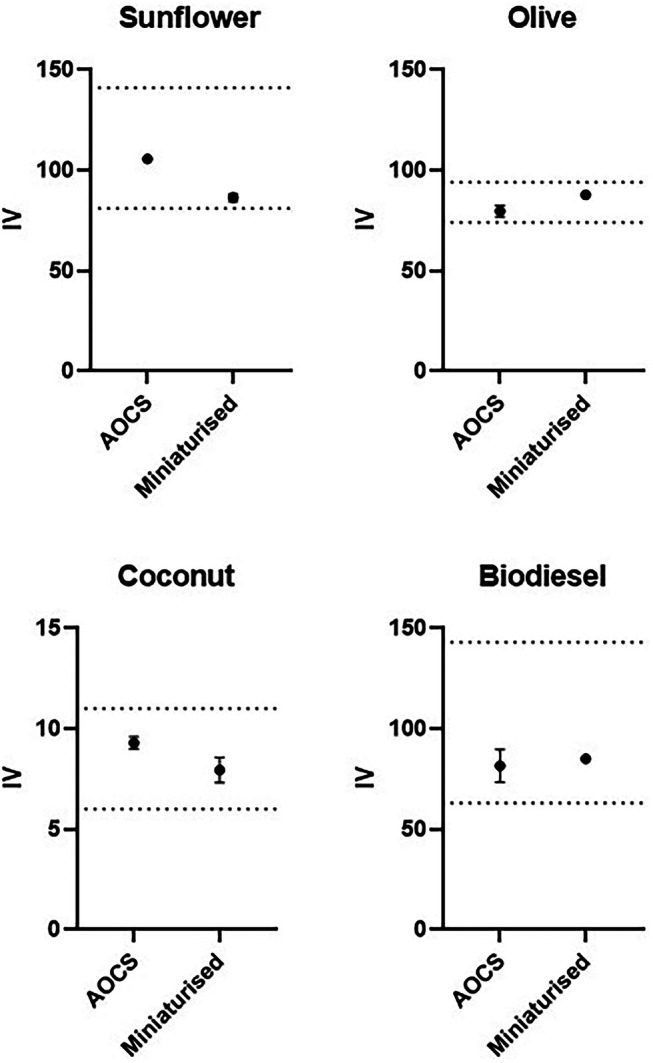
Comparison of the approved and miniaturized iodine value (IV) assay on natural oils and biodiesel. For sunflower oil, olive oil, and biodiesel, 0.5 g for the American Oil Chemists' Society (AOCS) method and 0.1 mg for the miniaturized method were used. For coconut oil, 2 g for the AOCS method and 1 mg for the miniaturized method were used. The dashed lines denote each sample's minimum and maximum value of the reported IV (*n* = 3, error bars correspond to the SEM).

To confirm the accuracy of the miniaturized IV assay across a range of biological lipid mixtures, sunflower oil, olive oil, coconut oil, and biodiesel were tested following both the AOCS protocol and the adapted miniaturized IV assay (Figure 4). When the expected IV is low (<10), a higher sample mass is recommended (AOAC 920.159) due to the scarcity of unsaturated bonds. Therefore, smaller amounts of sunflower oil, olive oil, and biodiesel were used than of coconut oil (Figure 4).

These results confirm that the miniaturized IV assay can measure the unsaturation of different lipid samples and report values in line with the literature. The volume of reagents needed and the time taken to perform the assay are considerably lower, especially when analyzing several samples, compared to the established titration method.

### Optimization of the miniaturized IV and detection of unsaturation in lipid standards

We then tested whether the miniaturized IV assay could be used on cell membrane lipids. Membrane fluidity is influenced by the length and proportion of saturated‐to‐unsaturated chain fatty acids in the lipid bilayer, the content and ratio of phospholipids, membrane proteins, cyclopropane fatty acid (CFA) and branched‐chain fatty acids (Qi et al., 2019), and, in eukaryotic systems, sterol content. Despite this complexity, various studies have demonstrated that the level of unsaturation of fatty acids is the principal factor determining membrane fluidity (Hagve, 1988) and can be modified by environmental factors (Linney et al., 2023) and disease states (Pan et al., 2021). In many cells, a higher proportion of unsaturated and short‐chain fatty acids in the cell membrane correlates with low‐temperature adaptation, where a more viscous membrane would otherwise be increased. In solvent‐producing cells, increased changes have been noted in the saturated fatty acid and CFA content, whereas unsaturated fatty acid content decreased within the cell membrane (Zhao et al., 2018). The proportion of unsaturated fatty acids also decreased with higher pH (Wu et al., 2012). Studying membrane adaptation, integrity, and fluidity mechanisms is critical for elucidating how cells maintain viability and regulate metabolic activities.

**FIGURE 5:**
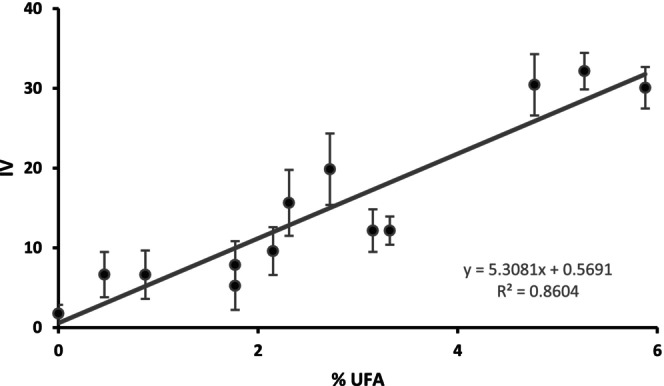
Linear regression showing the correlation between experimental iodine value (IV) and the average number of double bonds per fatty acid molecule (where %UFA is the percentage of unsaturated bonds per fatty acid, with 5.88% correlating with one unsaturation per fatty acid). The line of best fit equation and *R* ^2^ are shown.

Approximately 1 mg lipid sample was selected due to its minimal standard error and practicality as a sample size for a diverse array of biological samples when the degree of unsaturation is initially unknown. A 2 g wet‐weight cell pellet is typically sufficient to yield 3 mg lipid. Initially, assays were performed with mixtures of two defined lipids (18:1 and 14:0). By altering the ratio of these two standards, it is possible to determine the sensitivity of the assay while retaining the same total mass of lipid. The lipid mixtures used are shown in Table 1 and the linear trend between IV and unsaturation is clearly visible in Figure 5. This indicates that the assay is suitable for the detection of double bonds in defined lipid mixtures.

**TABLE 1:** Analysis of defined lipid mixtures using the miniaturized iodine value (IV) assay.

Lipid mixture (% w/w)	Theoretical IV	Experimental IV	Average double bond per fatty acid	Calculated average double bonds per fatty acid
100% 18:1 (*cis*) PC	32.29	30.08 ± 2.60	1.00	0.93 ± 2.60
90.9% 18:1 (*cis*) PC + 9.1% 14:0 PC	29.72	32.16 ± 2.29	0.91	0.98 ± 2.29
83.33% 18:1 (*cis*) PC + 16.67% 14:0 PC	27.54	30.45 ± 3.85	0.83	0.92 ± 3.85
60% 18:1 (*cis*) PC + 40% 14:0 PC	20.50	12.16 ± 1.77	0.60	0.36 ± 1.77
57.14% 18:1 (*cis*) PC + 42.85% 14:0 PC	19.61	12.16 ± 2.67	0.57	0.35 ± 2.67
50% 18:1 (*cis*) PC + 50% 14:0 PC	17.34	19.86 ± 4.48	0.50	0.57 ± 4.48
42.85% 18:1 (*cis*) PC + 57.14% 14:0 PC	15.02	15.64 ± 4.13	0.43	0.45 ± 4.13
40% 18: 1 (*cis*) PC + 60% 14:0 PC	14.08	9.59 ± 3.00	0.40	0.27 ± 3.00
33. 33% 18:1 (*cis*) PC + 66.67% 14:0 PC	11.85	7.85 ± 2.99	0. 33	0.22 ± 2.99
16.67% 18:1 (*cis*) PC + 83.33% 14:0 PC	6.08	6.63 ± 3.04	0.17	0.18 ± 3.04
9% 18:1 (*cis*) PC + 91% 14:0 PC	3.32	6.64 ± 2.84	0.09	0.18 ± 2.84
4.76% 18:1 (*cis*) PC + 95.24% 14:0 PC	1.77	5.24 ± 3.03	0.05	0.14 ± 3.03
100% 14:0 PC	0	1.75 ± 1.10	0	0.05 ± 1.10

*Note*: The table shows the composition of the lipid mixtures, the average number of double bonds per fatty acid, the theoretical and experimental IV, and the calculated average double bonds per fatty acid.

For each mixture, the theoretical IV was calculated (Equation 2; Hagve, 1988) and compared with the experimental IV of the mixtures (Table 1 and Figure 5). The IV was determined for each mixture and is denoted “Experimental IV” in Table 1. The mixtures were analyzed in triplicate.

Theoretical IV formula:

(2) $$\mathrm{IV}=\frac{2\times 126.92\times \mathrm{no}.\;\text{of double bonds}\times 100}{\text{molecular weight}}$$

The average number of double bonds per lipid molecule in each mixture was calculated using Equation (3) (Odoom & Edusei, 2015) and recorded in Table 1 as “calculated average double bonds per fatty acid.”

Calculation of the average number of double bonds per lipid molecule:

(3) $$\text{Average number of double bonds}\;\mathrm{per}\;\text{fatty acid}=\frac{\mathrm{IV}\times \mathrm{MW}\;\text{of substance}}{253.8\times 100\;}$$

Tests with representative defined lipid standards (22:6, 22:0, 18:1, and 14:0; Figure 6) were performed to confirm that the miniaturized IV assay could detect double bonds in cell membrane‐relevant lipids. Fifteen different lipid mixtures were prepared (Table 2 and Figure 7). The lipid mixtures ranged from 100% (w/w) 22:6 (*cis*) PC (6 average double bond per fatty acid) to 100% (w/w) 14:0, DMPC (0 average double bond per fatty acid). The latter was chosen as the blank because 14:0 is a saturated fatty acid with 0 unsaturation. The IV was obtained from all mixtures simultaneously using the miniaturized IV assay within a 96‐well plate. The whole procedure was performed in under 3 h.

**FIGURE 6:**
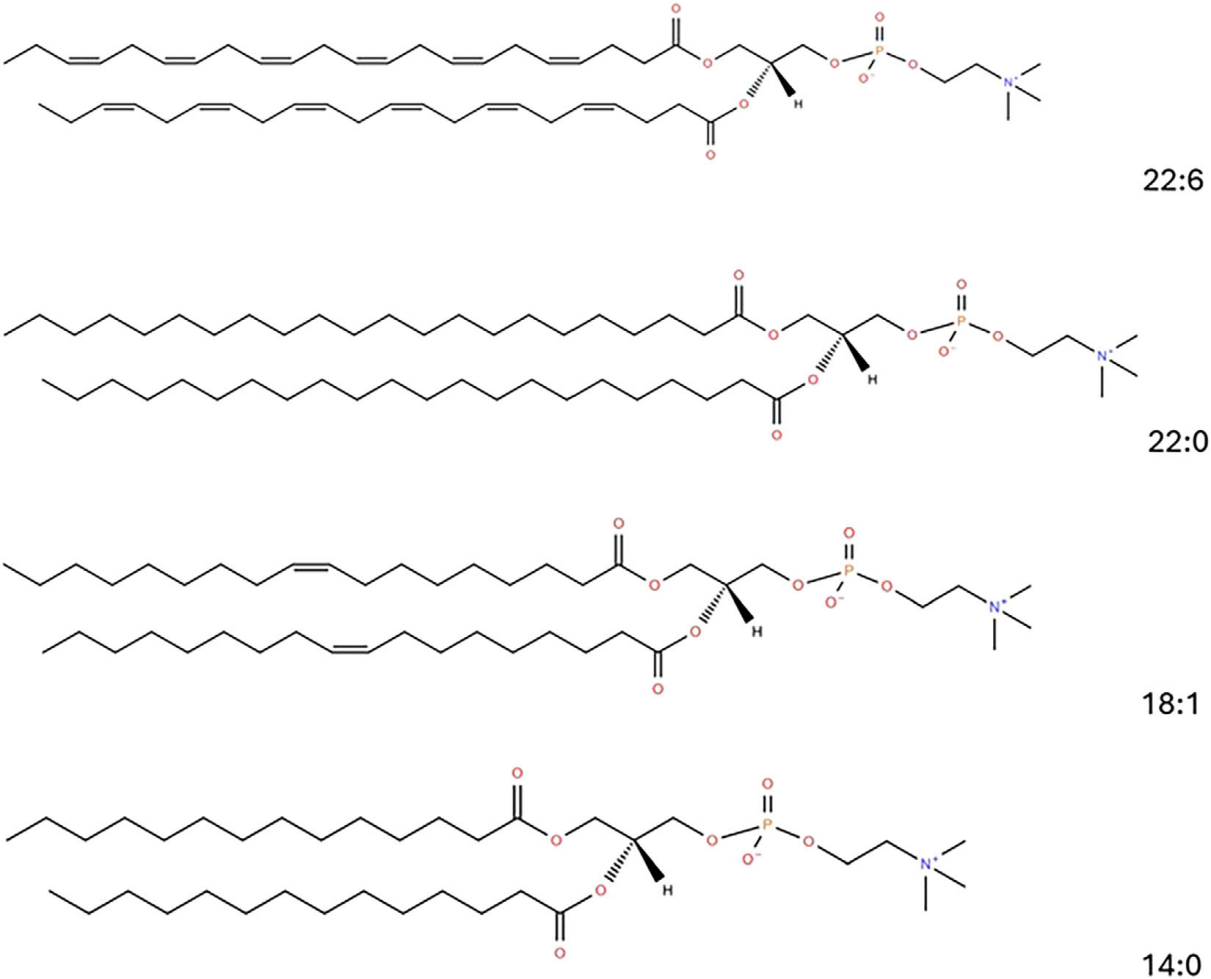
Structures of the lipid standards. The lipid standards used in the miniaturized iodine value assay were 22:6 (*cis*) PC, 22:0 PC, 18:1 (*cis*) PC, and 14:0 PC. Created with lipidmaps.org.

**TABLE 2:** Analysis of defined lipid mixtures using the miniaturized iodine value (IV) assay.

Lipid mixture (% w/w)	Theoretical IV	Experimental IV	Average double bond per fatty acid	Calculated average double bonds per fatty acid
100% 22:6 (*cis*) PC	173.43	152.35 ± 7.71	6.00	5.27
75% 22:6 (*cis*) PC + 25% 18:1 (*cis*) PC	141.00	141.77 ± 5.95	4.75	4.78
75% 22:6 (*cis*) PC + 25% 14:0 PC	137.94	130.08 ± 8.81	4.50	4.24
75% 22: 6 (*cis*) PC + 25% 22:0 PC	129.19	143.67 ± 14.72	4.50	5.01
50% 22:6 (*cis*) PC + 50% 18:1 (*cis*) PC	106.77	131.18 ± 5.56	4.00	4.30
50% 22:6 (*cis*) PC + 50% 14:0 PC	97.88	81.51 ± 11.43	3.00	2.50
25% 22:6 (*cis*) PC + 75% 18:1 (*cis*) PC	70.59	83.63 ± 10.16	2.25	2.67
25% 22:6 (*cis*) PC + 75% 14:0 PC	52.30	29.62 ± 10.6	1.50	0.85
25% 22:6 (*cis*) PC + 75% 22:0 PC	42.48	24.9 ± 5.05	1.50	0.88
100% 18:1 (*cis*) PC	32.29	32.31 ± 8.68	1.00	1
75% 18:1 (*cis*) PC + 25% 14:0 PC	25.08	40.89 ± 10.08	0.75	1.22
50% 18: 1 (*cis*) PC + 50% 22:0 PC	17.34	16.15 ± 8.94	0.50	0.47
50% 18:1 (*cis*) PC + 50% 14:0 PC	15.03	12.60 ± 4.42	0.50	0.42
25% 18:1 (*cis*) PC + 75% 14:0 PC	9.00	8.44 ± 6.81	0.25	0.23
100% 14:0 PC	0	Blank	0	Blank

*Note*: The table shows the composition of the lipid mixtures, the average number of double bonds per fatty acid, the theoretical and experimental IV, and the calculated average double bonds per fatty acid.

**FIGURE 7:**
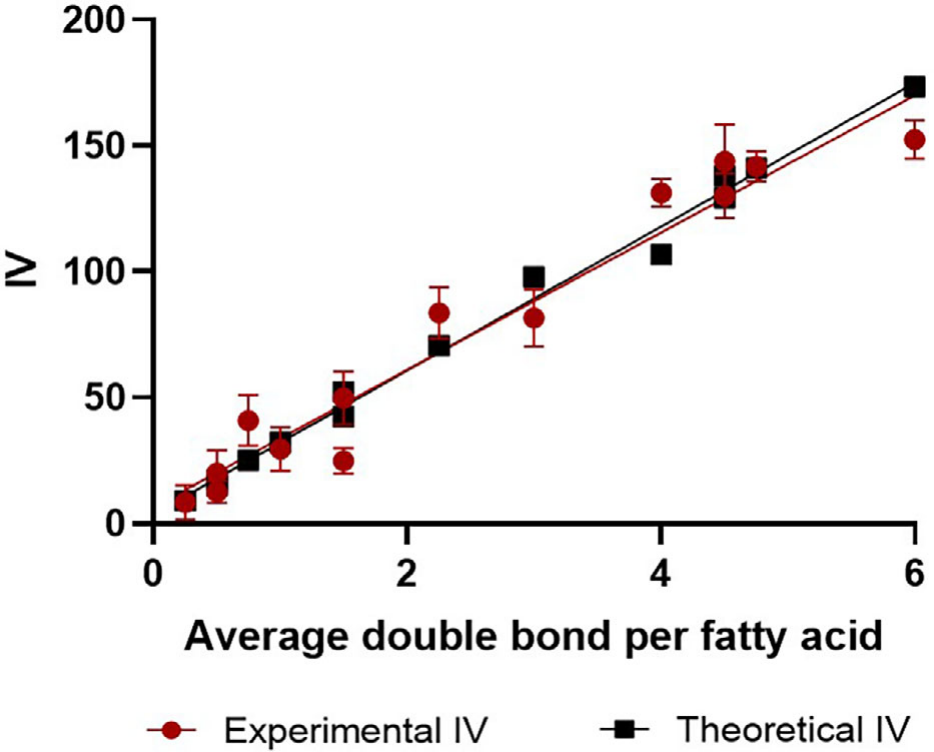
Quantification of iodine value (IV) for lipid standards. The black squares depict the theoretical IV of the standards, while the red circles represent the experimental values (*n* = 5, error bars correspond to SEM).

**FIGURE 8:**
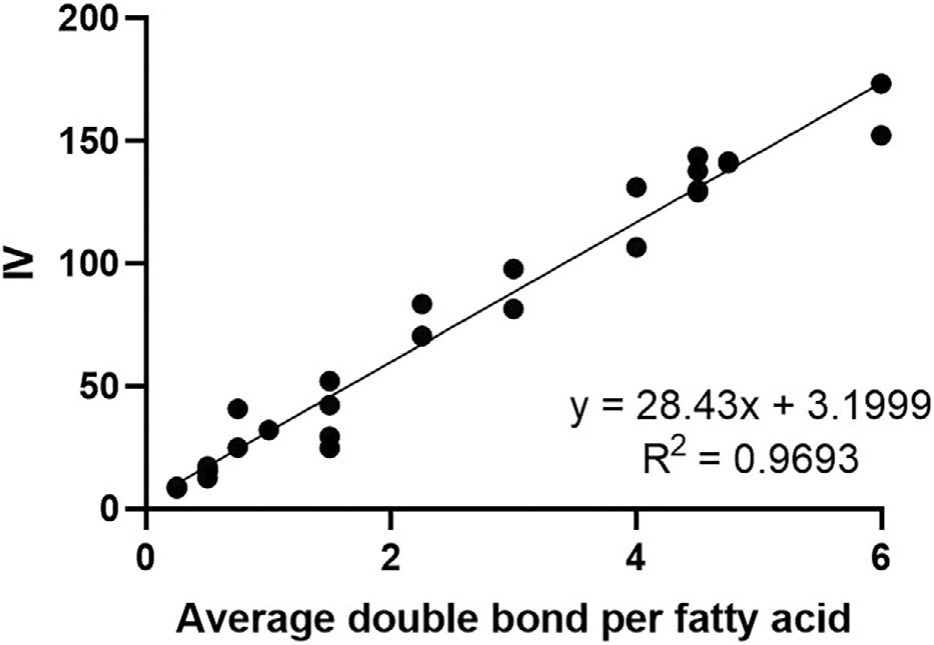
Linear regression showing the high degree of correlation between experimental iodine value (IV) and the average number of double bonds per fatty acid molecule. The line of best fit equation and *R* ^2^ are shown.

A linear regression model was used (Figure 8) to examine the correlation between the theoretical and experimental IV. With an *R* ^2^ = 0.9693, these results show that the correlation between the experimental and theoretical IV has a high confidence level. The sensitivity of the miniaturized assay enabled a mixture that contained an average of 0.25 double bonds per lipid molecule to be detected.

### Miniaturized IV assay analysis of *E. coli* cell membranes

**FIGURE 9:**
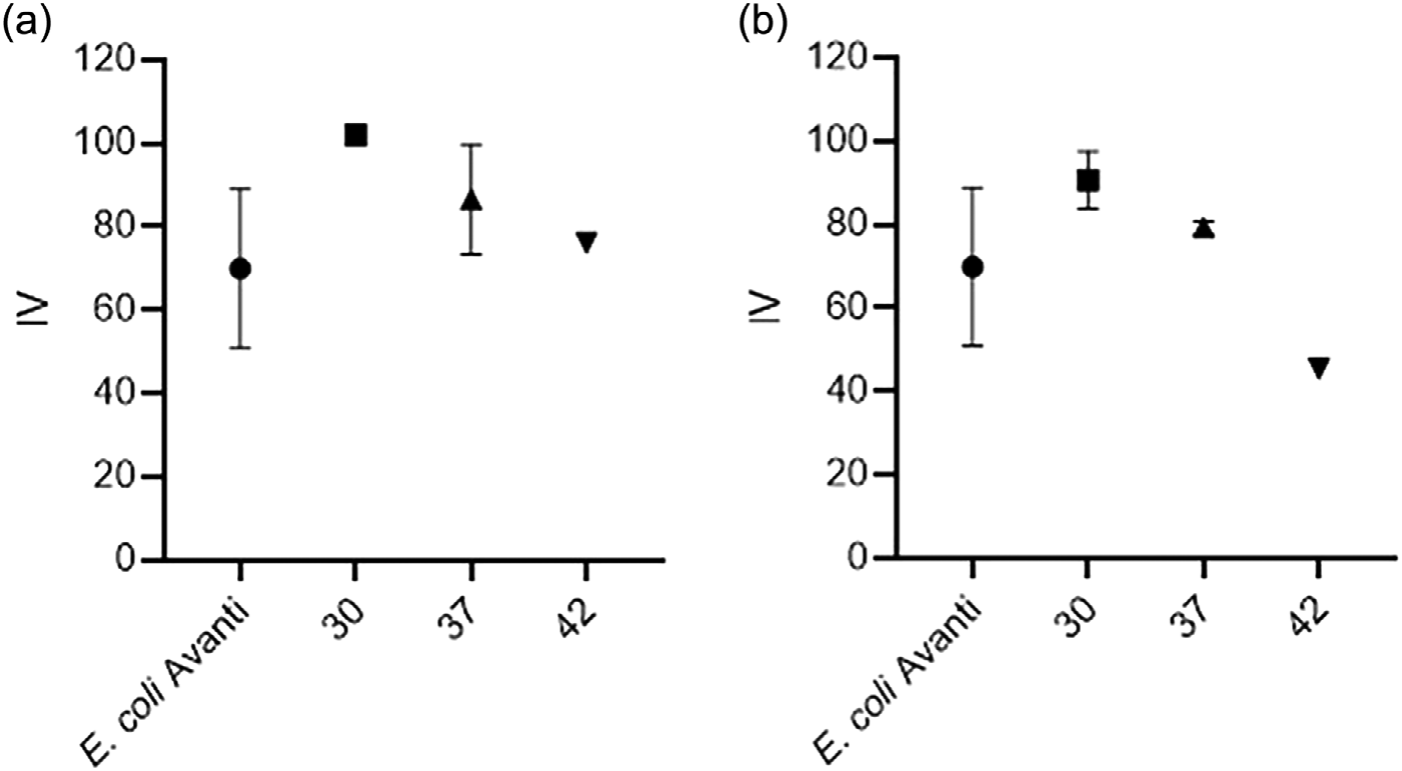
*Escherichia coli* miniaturized iodine value (IV) assay. The miniaturized IV assay was used on lipids from *E. coli* membranes grown at 30, 37, or 42°C. *E. coli* Avanti is the total lipid extract from Avanti (USA). 14:0 PC was used as a blank. Approximately 1 mg lipid was measured (*n* = 3, error bars correspond to the SEM). (a) The amount was calculated with the POPE:POPG:POPC 1:1:1:standard. (b) The lipids were calculated with the POPE:POPG:cardiolipin 1:1:1 standard.

The miniaturized IV assay was next applied to biological samples to demonstrate its utility. *E. coli* DH5α cultures were grown at 30, 37, or 42°C, and the lipids of the *E. coli* membrane were extracted using the Bligh and Dyer or the MTBE extraction method. We used the Stewart assay for lipid quantification (Stewart, 1980), which has several limitations, including poor detection of PC‐containing lipids. Consequently, we used two standard curves. POPE:POPG:POPC is a good lipid mixture that mimics samples when the content is unknown. It is relatively inexpensive and mimics the natural composition of biological membranes. POPE:POPG:cardiolipin is a good mimic of well‐characterized *E. coli* membranes, in ratios 1:1:1. Based on both curves, 1 mg lipid was used for the IV assay (Figure 9). Approximately 1 mg 14:0 DMPC lipid standard was used as the blank. As an *E. coli* standard (*E. coli* Avanti), the *E. coli* total lipid extract from Avanti (USA) was used. Each cell's macromolecular composition and energy requirement differ depending on the strain, temperature, environment, and growth rates. Published average double bonds per fatty acid in *E. coli* strains grown at 37°C range from 0.24 to 0.64 (Mavis & Vagelos, 1972). A trend can be observed where lower temperatures are associated with higher IV, correlating with an increase in unsaturation and a fluidization of the membrane.

No statistically significant distinctions were detected among the different temperatures. The *E. coli* standard from Avanti has known concentrations of *E. coli* FAs, corresponding to 0.85 average double bonds per FA. Based on the direct proportion between the IV and the number of double bonds in the sample, the average number of double bonds per FA at three different temperatures was determined using the rule of three. The IV for *E. coli* lipids from Avanti was 69.9 ± 18.9, which corresponds to 0.85 average double bonds per fatty acid. For *E. coli* grown at different temperatures, the IV values using the PC‐containing standards were 101.9 ± 2.3, 86.5 ± 13.1, and 76.1 ± 0.0 at 30, 37, and 42°C, respectively. This corresponds to 1.24, 1.05, and 0.93 of average doble bonds per FA. For CL‐containing standards, the IVs were 90.7 ± 6.9, 79.3 ± 1.53, and 45.4 ± 0.0 which correspond to 1.1, 0.96, and 0.55 average double bonds per fatty acid. It is well‐established that the cell membrane requires a reduced melting temperature at low temperatures to enhance membrane fluidity, which is crucial for proper membrane function. Bacteria have adopted a mechanism to achieve this by increasing unsaturated fatty acids proportionately (Bajerski et al., 2017). Using gas chromatography, Morein et al. (1996) determined the acyl chain composition of *E. coli* cultured at 37 and 27°C, obtaining an average double bond number per fatty acid of 0.47 and 0.54, respectively. Although the increase in double bonds was not statistically significant, their data indicated a trend toward higher unsaturation at lower temperatures. Similarly, the miniaturized IV assay shows a trend where lower temperatures increased saturation.

Modified IV methods are still being developed to make the assay cheaper, faster, and more accessible. The miniaturized IV assay presented here represents an improvement over current formats because of the minimum amounts of hazardous reagents required and waste generated and its applicability in quantifying unsaturation in fatty acids of cell membranes. The method uses a small amount of biological material (approximately 2.5 g of biomass). Conventional methods such as mass spectrometry and NMR require cell lysis and lipid extraction, involving solvents, making them a multistep procedure that can be time‐consuming. The miniaturized IV assay requires the same pre‐steps as the conventional methods. However, it offers the advantage that the rest of the procedure can be performed in a multiwell plate and can be semi‐automated, with the whole process taking under 3 h. If more granular detail is needed, the results obtained can be correlated with mass spectrometry using the same sample preparation. While it is possible that the assay could be affected by contaminants that also contain carbon‐carbon double bonds, this limitation also applies to other techniques that simply detect the degree of unsaturated bonds in a sample, and if purifications of lipids are carried out carefully, this should not be an issue. Purification of lipids away from other cellular components is relatively trivial, and we would encourage users to do this before undertaking any lipid analysis.

Furthermore, the IV data analysis involves simple mathematical calculations. Spectroscopic methods have the advantage that minimal sample preparation is needed, or in some methods like infrared spectroscopy and Raman spectroscopy in vivo, real‐time monitoring is possible. Nevertheless, they require standards, optimization, and calibration, and for validation of the assay, mass spectrometry will still be needed (Patel et al., 2019). The miniaturized IV assay does not require advanced equipment; the procedure and data analysis are very straightforward and performed in a reasonable time and provide a reliable estimation of unsaturation, making it widely applicable even in laboratories with limited resources. We hope the research community will find this useful.

## CONCLUSIONS

The AOCS IV assay has been miniaturized and performed in microplates using a 1 mg lipid sample. This adaptation is advantageous because the assay now uses minimum hazardous reagents, can be used on biological samples with multiple concurrent assays, and ensures both reproducibility and simplicity of implementation in various laboratory settings without specialized equipment. The data analysis involves simple mathematical calculations. The miniaturized IV method was used on *E. coli* cell membranes to determine the average number of double bonds per lipid molecule, yielding results correlating with reported values. The miniaturized IV method will be a valuable tool when determining the percentage of unsaturation in lipid membranes and other biological samples where lipid quantity and technical resources are limited.

## AUTHOR CONTRIBUTIONS

*Conceptualization*: Alan D. Goddard. *Methodology*: Maria Monserrat Roman‐Lara. *Validation*: Maria Monserrat Roman‐Lara, Alan D. Goddard, and Roslyn M. Bill. *Formal analysis*: Maria Monserrat Roman‐Lara and Katie J. Chong. *Investigation*: Maria Monserrat Roman‐Lara. *Resources*: Alan D. Goddard and Roslyn M. Bill. *Writing—original draft preparation*: Maria Monserrat Roman‐Lara. *Writing—review and editing*: Alan D. Goddard, Roslyn M. Bill, and Katie J. Chong. *Visualization*: Maria Monserrat Roman‐Lara. *Supervision*: Alan D. Goddard, Roslyn M. Bill, and Katie J. Chong. *Funding acquisition*: Alan D. Goddard and Maria Monserrat Roman‐Lara. All authors have read and agreed to the published version of the manuscript.

## FUNDING INFORMATION

Maria Monserrat Roman‐Lara acknowledges support from grant CONACyT (2018‐000024‐01EXTF‐00053). Alan D. Goddard and Roslyn M. Bill acknowledge BBSRC award BB/R02152X/1 as part of the project “MeMBrane – Membrane Modulation for BiopRocess enhANcEment,” which is embedded in the ERA CoBioTech action of the ERA‐NET Cofund under the European Union's Horizon 2020 research and innovation program. The Aston Institute for Membrane Excellence (AIME) is funded by UKRI's Research England as part of their Expanding Excellence in England (E3) fund.

## CONFLICT OF INTEREST STATEMENT

The authors declare that they have no conflict of interest.

## ETHICS STATEMENT

No humans or animals were used in this research.

## Data Availability

The data that support the findings of this study are available from the corresponding author upon reasonable request.
